# Calcium Alginate-Based Hydrogel-Encapsulated Nutrients and Nucleic Acid Delivery for Ameliorating Saline–Alkali Stress in Plants

**DOI:** 10.3390/gels12070592

**Published:** 2026-07-02

**Authors:** Muhammad Riaz, Lixia Li, Ping He, Rong Jiang, Yanmei Li, Wentian He

**Affiliations:** 1 Institute of Plant Nutrition and Environmental Resources, Beijing Academy of Agriculture and Forestry Sciences, No. 9 Shuguanghuayuan Midroad, Haidian District, Beijing 100097, China; muhamriazibnsajjad5@gmail.com (M.R.); ashleyllx@163.com (L.L.); jiangrong@baafs.net.cn (R.J.); 2 Beijing Engineering Technology Research Center for Slow/Controlled-Release Fertilizer, No. 9 Shuguanghuayuan Midroad, Haidian District, Beijing 100097, China; 3 Institute of Cropping Resources & Regional Planning, Chinese Academy of Agricultural Sciences, Beijing 100081, China; heping02@caas.cn

**Keywords:** calcium alginate, rheological behavior, mannuronic (M) and guluronic (G) acid, ionic crosslinking, smart bioencapsulation, sustainable crop

## Abstract

Calcium alginate is an anionic polysaccharide that forms an ionically crosslinked hydrogel network with encapsulation properties to nucleic acids and nutrients for the amelioration of osmotic stress, ion toxicity and nutrient imbalance in saline–alkali soils. Traditional soil reclamation methods, including salt leaching, incorporation of organic matter, and gypsum application, are water-intensive under a changing climate, ultimately necessitating transformative bio-based solutions for food security. Calcium alginate-based biohydrogel represents a versatile platform with a tunable macromolecular architecture, ionic crosslinking via an “egg box” mechanism and potentially promising to deliver engineered co-encapsulated nutrients and genetically modified cargoes. The mannuronic (M) and guluronic (G) acid (M/G) ratios govern ion exchange capacity, rheological behavior and release kinetics in saline- and alkali-stressed environments. Recent studies on alginate-based nutrient encapsulation showed reduced oxidative damage and a 15–50% increase in plant-available water. The irrigation intervals extended from 7 to 14 days and yield gains by 24% in wheat, with comparable improvements in maize, tomato, rice and cotton. Calcium alginate hydrogels encapsulated salt tolerance genes (*HKT1*, *SOS1*, *AVP1*) encoding proteins mainly involved in Na^+^ retrieval from xylem, Na^+^ extrusion from root cells and vacuolar Na^+^ sequestration, which have achieved yield gains of 40 to 75% across wheat, rice and maize. Future research should focus on optimizing mechanical strength, crosslinking chemistry and smart bioencapsulation strategies for sustainable development so that crops are capable of withstanding variable climate stresses.

## 1. Introduction

Calcium alginate is a naturally occurring anionic biopolymer, extracted from brown algae and found as a biofriendly macromolecule used to engineer multi-responsive biohydrogels [1]. Seaweed can be washed with an acidic source before being neutralized with Na_2_CO_3_ to produce water-soluble sodium alginate, which is then crosslinked with Ca^2+^, e.g., from CaCl_2_ solutions of various concentrations, to yield insoluble calcium alginate. This anionic linear polysaccharide made of M and G residues, which can give rise to hydrogels via the ionic crosslinking of alginate G-blocks (Chains), arises through the “egg-box” mechanism with the aid of divalent cations [2]. Calcium alginate addresses biotic and abiotic stresses through a twofold approach: delivering nutrients to maintain ion homeostasis under hostile ionic conditions, and encapsulating nucleic acids in a protective matrix to enhance yield [3]. The capability of hydrogel-coated systems to simultaneously retain nutrients and genetic cargoes, i.e., CRISPR-Cas9 ribonucleoprotein complexes, messenger RNA (mRNA), long non-coding RNA (lncRNA) and small interfering RNA (siRNA), positioned calcium alginate as a versatile platform to promote genetics and saline–alkali tolerance through trait engineering [4]. The diversity of calcium alginate-encapsulated genetic moieties, their target genes, delivery routes and reported performance outcomes are systematically summarized. The global degradation of soil is increasing, especially in saline and alkaline soils, which limit the productivity of about 1.5 billion ha of land in the world as a result of industrialization, resource depletion and population growth. It is estimated that every year, an average of 7–12% of the affected area is being expanded due to climatic aridification, rising sea levels, and salt water encroachment [5]. The problem of secondary salinization is undermining approximately one-third of the irrigated land in the world, exacerbating food security issues in regions that are already exposed to challenges of water and land scarcity. Soluble salt deposition at the field scale decreases soil water potential, leading to osmotic stress, poor uptake of root water and increased Na^+^, Cl^−^, CO_3_^2−^ and HCO_3_^−^ ions, reducing ionic homeostasis and damaging plasma membrane integrity [6]. These impacts are cascaded by ionic toxicity, nutrient imbalance, oxidative stress and photosynthetic inhibition, which, in combination, lower yields in glycophytic crops that are sensitive to salt, including rice, wheat, maize and tomato. Conventional reclamation methods (i.e., salt leaching, gypsum amendment, drainage enhancement and organic matter addition) are less appropriate and requires vast amounts of good quality water, a resource whose availability is increasingly erratic under a changing climate [7]. Equally, traditional breeding programs focusing on salt-tolerant cultivars are limited by the restricted genetic variation and an 8- to 12-generation selection process prone to polygenic complexities of salt tolerance characteristics. This dual insufficiency highlights the need for combined technologies capable of alleviating soil stress while reinforcing the inherent genetic capacity of plants to withstand saline and alkaline conditions. Calcium alginate hydrogels are formed via ionic gelation, in which sodium alginate crosslinks with divalent Ca^2+^ ions binding to guluronic acid (G) blocks through the “egg-box” supramolecular structure [8,9,10,11]. The Ca^2+^ ions are chelated in the electronegatively linked G residues of antiparallel chains, with each cation chelated to 9 or 10 oxygen atoms forming junction zones by aligning in parallel with G-blocks. This architecture is able to tune mesh porosity from nanometers to micrometers with a polymer M/G ratio and density of crosslinking to control ion exchange capacity of ~0.5 mmol g^−1^, alongside dynamic swelling kinetics, controlled release of nutrients and macromolecular genetic cargoes [12]. A multiscale characterization framework is outlined, which details the types of techniques required to resolve the structure, function and encapsulation relationships across nine orders of magnitude in length scale. Composite hydrogels with cellulose nanofibers, carboxymethyl cellulose (CMC) and chitosan improve mechanical stability and show stimuli-responsive characteristics with a change in their release profile of pH and ionic strength reaction [13]. The anionic backbone enables nutrient encapsulation and renders calcium alginate an efficient nucleic acid carrier. The negatively charged genetic cargoes are loaded using cationic bridging reagents, e.g., chitosan, poly-L-lysine, polyethyleneimine, which condense nucleic acids into nanocomplexes and embed them within the hydrogel shell matrix, shielding the cargo from nuclease degradation [14]. Alginate-encapsulated fertilizers integrated into one important platform to buffer the ionic toxicity and leaching of nutrients were reduced by 30 to 40% [15]. Meanwhile, CRISPR-Cas9 complexes under the protection of the alginate allow for the editing of salt tolerance genes, e.g., *HKT1*, *SOS1*, *AVP1*, without DNA damage or off-target effects. The encapsulated mRNA allows the expression of enzymes for transient expression and siRNA constructs silence negative salt tolerance regulatory elements without long-term genomic integration and specialized tissue culture facilities. The innovations described here include improvements in the calcium alginate molecular structure, crosslinking chemistry and functional properties of copolymerized composite hydrogels [16]. Calcium alginate formulations enable agriculturists and agronomists to overcome short-term edaphic constraints with long-term genetic predisposition, which opens the door for sustainable food production on marginal land that may not be possible via other soil or genetic modifications. There are research implications, including (1) the effects of crosslinking density, M/G ratio, ion selectivity on biomaterial encapsulation of nutrients and nucleic acids [17]; (2) rate of alginate degradation in the soil microbial habitat [15]; (3) the relationship between molecular weight, rate of swelling and drying; (4) chemical stability in varying pH conditions [18]; and (5) biosafety and regulatory concerns for non-biofriendly coating proportions. In this review, we shed light on (i) plant and soil stress pathways in saline–alkali conditions; (ii) alginate-based hydrogel classification by source, macromolecular structure and crosslinking mechanism [19]; (iii) ameliorative mechanisms of hydrogels to reduce osmotic, ionic and oxidative stress in plants; (iv) nutrient encapsulation and micronutrient delivery of hydrogels for improved nutrient use efficiency in stress conditions; (v) nucleic acid coatings and delivery systems, i.e., CRISPR-Cas9, mRNA/lncRNA and siRNA/RNAi, for crop resilience and food security [20].

## 2. Plant Stress Responses in Saline and Alkaline Soil

**Figure 1:**
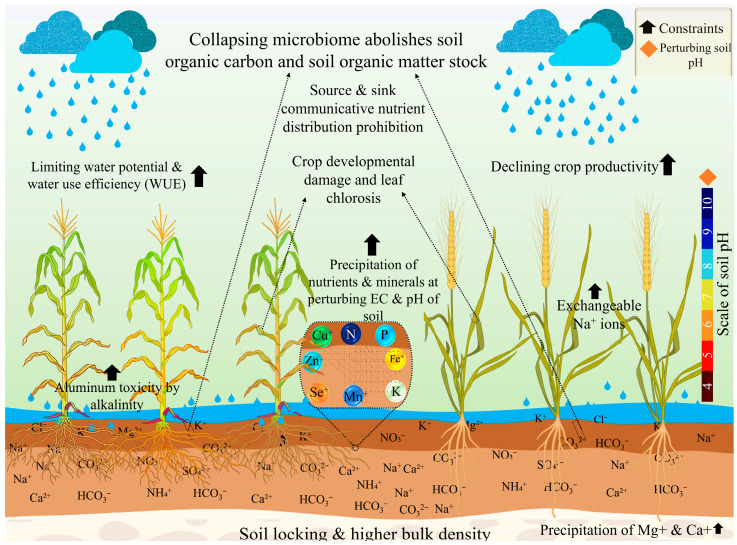
Effects of soil salinization and alkalinity on crops. The excessive salts and changes in pH disrupt nutrient availability, destabilize the soil microbiome, decrease water use efficiency and slow growth. Increasing soil bulk density and ion imbalances eventually leads to decreasing agricultural productivity in the saline-sodic stress conditions.

The accumulation of soluble salts and alkalizing ions in the rhizosphere creates interlinked limitations to crops, including osmotic limitation, ion toxicity and nutrient imbalance, directly limiting crop growth (Figure 1) [21]. Plant oxidative stress is caused by impaired electron transport, which results in the excessive production of reactive oxygen species (ROS), including H_2_O_2_, O_2_^−^ and lipid peroxidation, observed as increased malondialdehyde (MDA) levels. Photosynthetic apparatus is highly susceptible to osmotic stress and stomatal closure, which reduces CO_2_ supply. This leads to the degradation of photosystem II (PSII), thylakoid membranes and chlorophyll pigments, decreasing quantum efficiency (Fv/Fm) and biomass formation [22]. Na^+^ competes with K^+^ along transport pathways, while alkaline conditions cause phosphate precipitation and depletion of Fe, Zn, Mn, and other trace micronutrients, inhibiting chlorophyll function and enzyme activity, thereby causing nutrient imbalances. High levels of Na^+^ and Cl^−^ ions perturb K^+^ binding affinity and break the protein structure. Higher levels of pH, CO_3_^2−^ and HCO_3_^−^ replace Ca^2+^ and Mg^2+^ ions that destabilize the adhesion of the plasma membrane, leading to further instability in ionic homeostasis [23]. Such ionic imbalances reduce soil water potential and decrease the hydraulic gradient that drives root water uptake. Consequently, root turgor pressure decreases, shutting down stomata, and CO_2_ assimilation decreases. Alkaline stress further decreases both water potential and water use efficiency (WUE), resulting in ionic toxicity (Na^+^, Cl^−^), alongside alkalinity-enhanced aluminum toxicity, which severely limits root function [24,25]. In such circumstances, plants shift their energy to the osmotic adjustment process by storing proline, betaine and soluble sugars. The decline in rhizosphere microbiomes disrupts the cycling of organic carbon and organic matter in the soil, hindering the source sink communication and nutrient allocation of plant system [26,27]. This further undermines long-distance water transportation through the soil–plant–atmosphere hydraulic impairment. This involves a reduction in aquaporin activities and root–stem conductance, further undermining long-distance water transport. Plants respond to salt–alkali stress through the concerted activation of a suite of enzymatic and non-enzymatic defense systems. It includes the plasma membrane-based Na^+^-efflux transporter *SOS1*, vacuolar Na^+^/H^+^ antiporters (*NHX*), and ROS-scavenging enzymes such as catalase (CAT) and superoxide dismutase (SOD). Moreover, low-molecular-weight antioxidants such as phenolic compounds and ascorbate are also critical players for this mechanism. Continuous stress leads to progressive breakdown of these homeostatic processes, resulting in ionic toxicity and oxidative damage to the plant’s developmental machinery [28,29]. Recent studies by Popova et al. (2023) [30] and Athanasiou et al. (2025) [31] have shown that soil-immobilized hydrogels effectively mitigate such detrimental effects in salt-stressed species like *Pisum sativum* and *Solanum lycopersicum*. It is evidenced by reduced hydrogen peroxide (H_2_O_2_) and malondialdehyde (MDA) levels and the re-establishment of cellular redox balance and membrane integrity. Plants exposed to the hydrogel treatments showed restoration of the transmembrane potential and growth rates similar to control plants. These data furnish compelling physiological, ionic and molecular genetic evidence for the efficacy of external interventions and highlight calcium alginate biohydrogel as a promising candidate for further mechanistic studies for agronomy [11,32].

## 3. Chemical Structure and Type of Alginate Hydrogels

**Figure 2:**
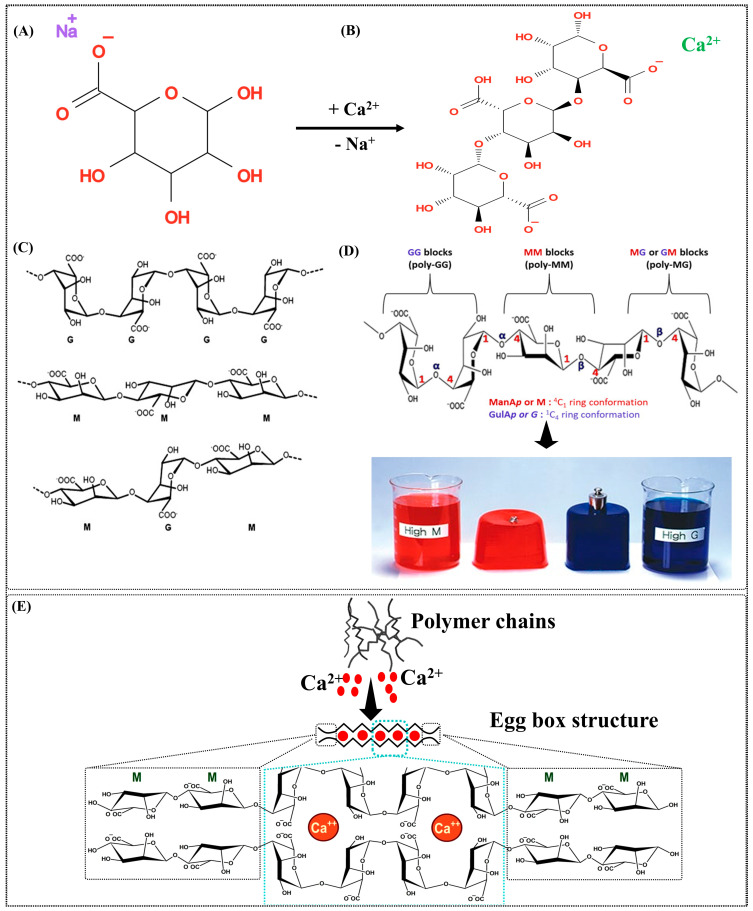
Alginate biopolymer macromolecular structure and gelation. (**A**) The structure of sodium alginate. (**B**) The structure of calcium alginate. (**C**) Heteropolymeric and homopolymeric guluronate (G)/mannuronate (M) of alginate, reproduced from [34] licensed under (CC BY 4.0). (**D**) Alginate block structures showing M (^4^C_1_, soft/flexible) and G (^1^C_4_, hard/rigid) conformations. M/G ratio determines gel mechanical properties, adapted from [35] licensed under (CC BY 4.0). (**E**) The “egg-box” structure (purple dotted line highlights and left–right dotted box represent the polymer chains) representation of calcium-mediated crosslinked chains of G-block polymer, forming a stable hydrogel network, adapted from [10] licensed under (CC BY 4.0).

The chemical structure of alginate is a linear unbranched anionic polysaccharide made up of two C-5 epimeric uronic acid residues: β-D-mannuronic acid (M) and α-L-guluronic acid (G). The carboxylate groups in the polymer chain are compensated by Na^+^ ions when they are in the form of a water-soluble salt (sodium alginate) (Figure 2A) [33]. The addition of divalent calcium ions (Ca^2+^) to the solution replaces Na^+^ ions and links neighboring chains together to form a crosslinked polymer, which is insoluble calcium alginate (Figure 2B). These residues are organized in homopolymer blocks in alternate GG or MM residues successively by heteropolymer alternating sequences (MG/GM) (Figure 2C) [34]. These segments in terms of proportion, sequential distribution and average block length together in the form of the M/G ratio are the basic determinants of the macromolecular conformations. The chain rigidity and gelation behavior of the polysaccharide are also important parametric features in this determination. The buckled, 2 → 1 helical ribbon-like structure of G-blocks is due to the 1 → 4 diaxial glycosidic linkage of guluronate residues that confer significant stiffness to the chains and produce a chain of electronegative cavities along the polymer. In contrast, M-blocks take a longer and more flexible 1 → 4 di-equatorial conformation, whereas alternating MG segments have intermediate flexibility (Figure 2D) [35]. This conformational alteration is the basis for the molecular foundation in the alginate variable gelling potential. It is only the rigid buckled G-block motifs which have the fine geometry necessary to organize divalent cations and form gel junction domains, validated experimentally by Solution NMR (M/G ratio, NG > 1) and FTIR (COO^−^ crosslinking index), as detailed in Table 1 and Table 2. The calcium alginate (Figure 2B) crosslinking process can be characterized by the “egg-box” (Figure 2E) model, originally proposed by Grant et al. (1973) and later optimized using X-ray fiber diffraction with circular dichroism and molecular dynamics measurements [36]. In this model, a pair of antiparallel chains in the GG-block align in register and divalent cations. In this model, Ca^2+^ ions are chelated in the diamond-shaped cavities between the paired chains. Oxygen atoms of the carboxylate, hydroxyl and ring oxygen functionalities of four guluronate chains coordinate each Ca^2+^ ion. It creates a nine- or tenfold oxygen coordination environment, which incorporates bound water molecules into the inner coordination geometry. In this process, initial Ca^2+^ binding triggers a conformational tightening that reorganizes nearby G residues for subsequent capture by additional cations, which further propagate along paired GG-blocks in a zipper-like fashion to create extended junction regions. The resultant dimeric egg-box junctions also associate, whereas other chains of alginate can be added onto the existing dimers to create multichain aggregated bundles. The conformational arrangement mechanism increases the thickness of junction zones, gel rigidity and brittle strength [36,37] (Figure 2A–E). Ca^2+^ concentration controls the extent of lateral aggregation, G-block length and concentration of the polymer, offering several parameters to control gel architecture, which can be quantified via XRD (d-spacing, CI), SAXS (mesh size ξ, persistence length Lp), and CD spectroscopy (molar ellipticity) (see Table 1 and Table 2). The “egg-box” mechanism is ion selective in the following order, i.e., Ba^2+^ > Sr^2+^ > Ca^2+^ and Mg^2+^, and suggests its steric complementarity of the cation size to the interchain chelation cavity [11]. The Mg^2+^ is not suitable to bridge the two GG chains, and gelation does not occur, but the Ca^2+^ has an ideal balance of binding. The biological safety and effective application of Ca^2+^ to agricultural, polymer and biomedical context make it the preferred crosslinker. The aggregation level of egg-box junction zones in the gel network controls key parameters, such as a denser junction density giving a tighter mesh with a smaller effective pore size, typically 5 to 20 nm, and higher elastic modulus with slower diffusional release [38,39]. The less dense junction density gives a more porous and swellable network, with pore dimensions up to several micrometers. The mesh sizes in the desired range are determined by swelling (Flory–Rehner ξ) and Micro-CT (porosity, connectivity) measurements, as shown in Table 2. Tunable porosity makes calcium alginate a versatile carrier of cargo molecules with a wide range of molecular weights, spanning several orders of magnitude, including small inorganic nutrient ions to complex macromolecular nucleic acids. All of these depend on the polymer concentration, M/G ratio, G-block length and crosslinking cation concentration [40]. The inherent characteristics of this macromolecular architecture make it desirable to incorporate within the core and matrix. The surface coats a wide array of bioactive substances in nature, size of nutrients and nucleic acids. The contributors to the dense anionic charges are deprotonated carboxylate groups of the alginate backbone that remain ionized at near-neutral pH (pKa of mannuronic acid ≈ 3.38; guluronic acid ≈ 3.65) and contribute to electrostatic complexation with cationic nutrients, e.g., NH_4_^+^, K^+^, Ca^2+^, Zn^2+^ and Fe^2+^/Fe^3+^, to enable efficient encapsulation within the gel matrix [41]. Their binding stoichiometry measured by ICP-OES (exchange capacity, meq/g) and ion-selective electrodes (partition coefficient Kd) is discussed in Table 2. The adjustable-size pores additionally permit physical entrapment of nutrients, nucleic acid, slow-release fertilizer particles and microbial inoculants inside the hydrogel core. Negatively charged nucleic acid molecules do not form direct interactions with the anionic alginate matrix due to electrostatic repulsion [8,42]. This is addressed by the use of intermediary cationic bridging agents like chitosan, poly-L-lysine or polyethyleneimine (PEI), which electrostatically condense the nucleic acid cargo into positively charged nanocomplexes, which are then trapped in the alginate gel during ionic gelation. Alternatively, cationic polymer and alginate coatings can be deposited in layers around pre-existing nucleic acid nanoparticles to give a core–shell architecture with a well-defined thickness of the coating and surface charge and degradation rate [39]. The hydrogel shell offers a protective microenvironment that protects encapsulated nucleic acids against degradation by extracellular nuclease, pH-induced hydrolysis and mechanical shearing within the soil and during foliar delivery. The stimulus-sensitive swelling characteristics of the hydrogel allow a triggering response and site-specific release against environmental factors, such as pH fluctuations or ionic gradients in the rhizosphere. Further, the soft and aqueous phase ionic gelation conditions by which calcium alginate hydrogels are typically formed at room temperature and near-neutral pH in the absence of organic solvent [43,44]. Moreover, it preserves the structure and function of thermally or chemically sensitive biological molecules, which is particularly beneficial for sensitive RNA cargoes. The tunability of shell thickness, mesh size, pore structure and surface modifications makes calcium alginate an ideal system to encapsulate, retain and deliver nutrients and nucleic acids in a single functional platform [45,46].

## 4. Classification and Crosslinking of Biohydrogel

**Figure 3:**
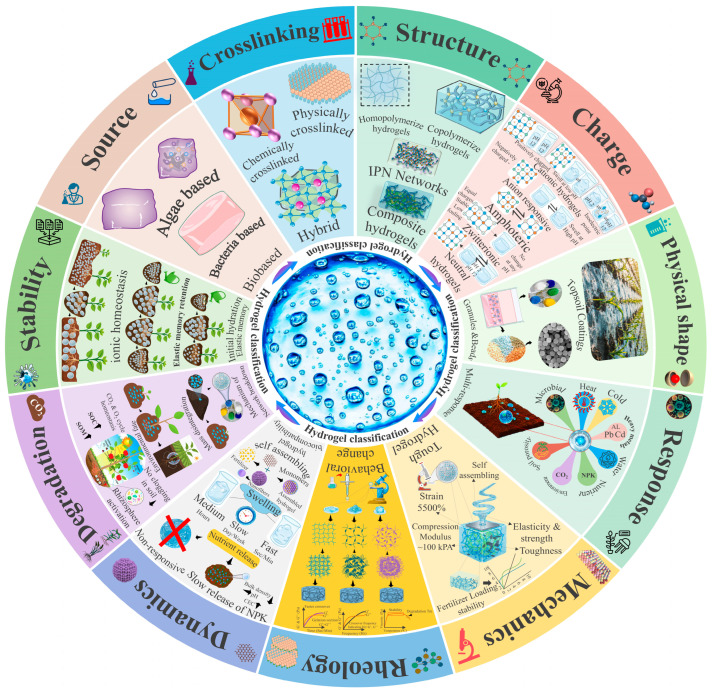
Generalized classification of biohydrogels for sustainable agriculture. Multidimensional radial diagram is a hydrogel classification that relies on crosslinking, structure, charge, physical shape, stimuli response, mechanics, rheology, swelling dynamics, degradation, stability, source and behavioral properties. Summary shows wide range of design parameters used to control functionality of hydrogel, i.e., nutrient release kinetics, mechanical strength and biodegradability of hydrogel.

Smart alginate-originated hydrogels are versatile soil modifiers, capable of mitigating osmotic stress, reducing ionic toxicity, increasing nutrient use efficiency (NUE), maintaining soil structure and stimulating the rhizosphere microbiome, resulting in improved crop yield [68,69]. Hydrogels are primarily derived from algal and bacterial sources, with defining attributes, including rheology, crosslinking, charge, structure, degradation and swelling (Figure 3). The polymer matrix serves as an in situ microscale water reservoir with equilibrium absorption ratios of 500–1800 g g^−1^ [70]. Hydrogels take up atmospheric moisture during the dark hours, high humidity and release it into the rhizosphere for evaporative demand in daytime hours, thus regulating daily changes in ionic strength and maintaining soil–root and leaf water status. This buffer system increases the irrigation time from days to several weeks without causing wilting, even in poor, harsh and leached soils. Salt-resistant formulations based on carboxymethyl cellulose (CMC) can sustain swelling ratios of up to 560 g g^−1^ in NaHCO_3_/Na_2_CO_3_ conditions [71,72].

The pH-responsive nanocellulose vinylcaprolactam formulations have been shown to be functional in highly alkaline brines. This confirms their applicability for effective saline media validated by swelling ratio, Q (Flory–Rehner model) and oscillatory rheology (G’) and tan (δ), see Table 2. Polyelectrolyte hydrogels, such as alginate, carboxymethyl cellulose and sulfonated polymer, include high densities of anionic functional groups, mainly carboxylate (–COO^−^) and sulfonate (-SO_3_^−^). These can immobilize free toxic Na^+^ and carbonate species (CO_3_^2−^, HCO_3_^−^) in the rhizosphere through chelation and ion exchange. The positive ion exchange capacity with a value of about 0.5 mmol g^−1^ is of paramount importance in replenishing the cytosolic K^+^/Na^+^ ratios in plant roots [73]. The surface to core exchange gradient is mapped by XPS, ToF-SIMS and EDS, as indicated in Table 2. Furthermore, calibrated calcium alginate reduces percolation and deep drainage, increases water holding capacity and maintains moisture in the root zone of fine sandy soil. Hydrogel incorporation increases local moisture potential, reduces evaporation, and extends irrigation intervals from 7 to 14 days in structurally degraded soils. The local moisture enhancement supports hydraulic and reduced aquaporin activity and root stem conductance [74]. Cellulose-based multi-stimuli and lower critical solution temperature (LCST)-responsive nutrient and pH-sensitive ammonium release mechanisms containing hydrogels are useful in sodic soils. The functional release performance can be quantified via in vitro release (Korsmeyer–Peppas (n)), PFG-NMR (tortuosity τ), and compression testing (Table 1 and Table 2). The field-level application reduces nutrient leaching by up to 40%, equivalent to plant-available nitrogen, phosphorus and potassium, with a 22% increase in wheat grain yields in saline conditions. The swollen hydrogel particles increase the porosity, aggregate stability and reduce bulk density of the bulk soil by 7–29% in sandy soil, facilitating root penetration and aeration [75,76]. Plants treated with hydrogel have continually less proline accumulation and moderate antioxidant enzyme activities than untreated controls. This suggests that the hydrogel-based amendments lower the true stress intensity compared to eliciting increased defense response. The decrease in external osmotic and ionic pressure enhances plant intrinsic defenses, such as Na^+^ ion extrusion pumps (*SOS1*), tonoplast antiporters (*NHX*), compatible solutes accumulation, and enzymatic antioxidants such as catalase (CAT) and superoxide dismutase (SOD). Hydrogel action in sustaining rhizosphere moisture and ion equilibrium optimizes photosystem II (PSII), sustaining photosynthetic efficiency and pigment levels [77]. Thus, calcium alginate-based biohydrogel-based systems operate in three integrated ameliorative and delivery regimes, e.g., (i) nutrient and nucleic acid [15,78] and other moieties encapsulation and delivery; (ii) maintaining water balance, sustaining photosynthetic efficiency and oxidative damage at the plant level [79]; and (iii) regulating soil water activity, ion diffusion, nutrient leaching and nutrient availability at the soil level [80]. The sustainability of these biomaterials has been demonstrated through the use of biodegradable polymers and composites such as cellulose and alginate–nanocellulose complexes. These complexes can then be broken down to innocuous oligomers in weeks to months, eliminating the safety issues associated with microplastic buildup. However, there are several sustainability gaps to be addressed, such as the fatigue and stability of hybrid hydrogels during prolonged wet–dry cycles and pH changes, which are yet to be understood [81]. There is a lack of quantitative analyses related to hydrogel characteristics, such as crosslinking density and charge density to in situ ion exchange capacity. In addition, detailed life cycle analyses (LCA) of biodegradable and hybrid hydrogels are lacking, which highlights the need for the development of cost-effective and biofriendly encapsulations. Large-scale manufacturing processes for such formulations including modified protected nucleic acid coatings and biobased fertilizers are needed to address global food demands in a changing environment [82].

## 5. Calcium Alginate Hydrogel Nutrient Encapsulation and Mechanism of Action

**Figure 4:**
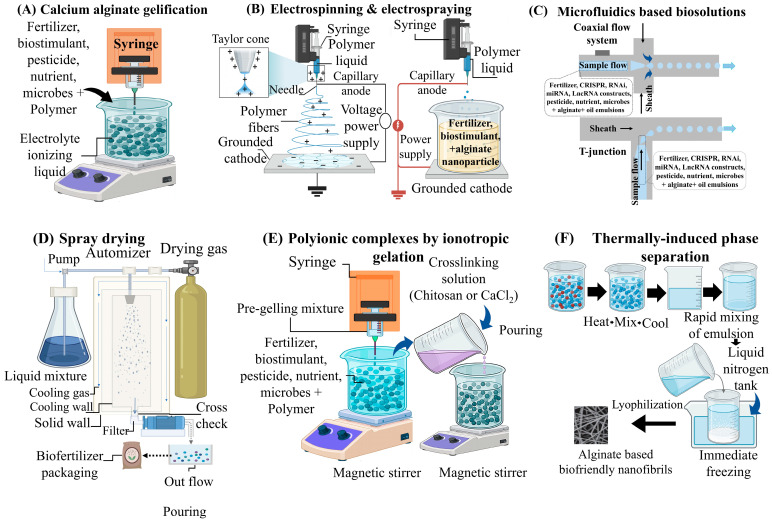
Approaches of alginate encapsulation systems. (**A**) Ca^2+^ alginate gelification. (**B**) Electrospinning and electrospraying. (**C**) Biosolutions using microfluidics. (**D**) Spray drying. (**E**) Ionotropic gelation of polyionic complexes. (**F**) Thermally induced phase separation to produce biofriendly nanofibrils. Those methods allow precise encapsulation and delivery of fertilizers, pesticides, bio-stimulants, nutrients, microbes, and genetic materials (CRISPR, RNAi, miRNA) to promote precision food security.

Hydrogels serve as encapsulator and synchronized nutrient release systems tailored for crops to achieve 30 to 40% reductions in nutrient leaching. Saline- and alkaline-tolerant calcium alginate biohydrogels function as an integrated platform that simultaneously address water scarcity, nutrient deficiency, soil reclamation, ionic toxicity and osmotic stress. The success of these systems depends critically on the encapsulation technique, which dictates bead morphology, loading efficiency, size distribution, and release kinetics. A number of fabrication techniques have been developed for calcium alginate-coupled nutrient carriers (Figure 4A–F) [79].

**Figure 5:**
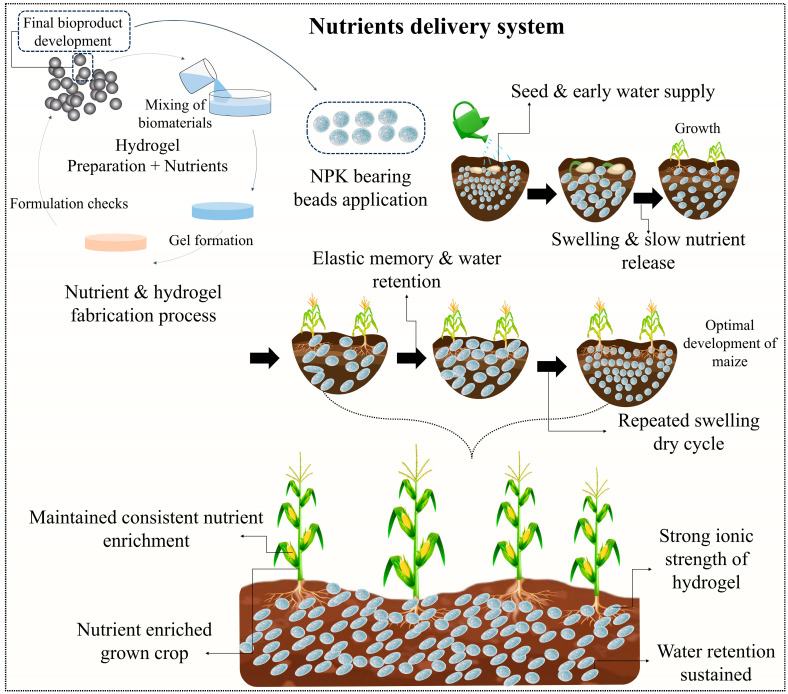
Hydrogel-based nutrient delivery system to crops and water conservation. The NPK-impregnated hydrogel beads are added to the soil and subjected to repeated swell–dry cycles, which allow for nutrient release, elastic memory water retention and ionic strength stability. This system provides long-term nutrient fortification and constant availability of moisture during crop season and production.

Extrusion dripping is the dropwise addition of sodium alginate urea solutions to a CaCl_2_ crosslinking bath. This method results in the formation of spherical beads of 0.5–5 mm diameter, the size of which is regulated by the nozzle gauge, flow rate, concentration of alginate and gelation bath. Morphological confirmation can be made by SEM/Cryo-SEM (pore diameter, wall porosity) and CLSM (3D cargo localization), see Table 1 and Table 2. Sodium alginate solutions (2–4% *w*/*v*) pre-impregnated with urea in loading ratios of 1:1 to 1:4 is extruded into 0.1–0.3 M CaCl_2_. In this method, urea is entrapped within the core-forming matrix. The direct relationship between shell density and core porosity is the duration of crosslinking from 5 to 30 min [83]. Shorter gelation duration develops softer beads, and a longer crosslinking time leads to slower burst release; denser beads bring diffusion-regulated release of 15 to 30 days. Further control of pore architecture is achieved post-gelation through air or freeze drying [84]. The freeze-dried beads rehydrate quickly upon soil contact, and air-dried beads swell slowly to postpone the onset of release. Internal gelation forms smaller microspheres of 50 to 500 µm that can be used in seed coating, whereas spray drying and electro spraying can form sub-100 µm microparticles on an industrial scale, but inlet temperatures above 120 °C can cause urea decomposition [85]. The generation of droplets in microfluidics can attain monodisperse beads (coefficient of variation <5%), allowing accurate study of release kinetics relationships with bead geometry. Coatings of urea granules by fluidized bed spraying onto single-layer alginate solutions produce conformal shells of 20–200 µm thickness that slow down dissolution. These can be susceptible to premature rupture when subjected to osmotic pressure and mechanical abrasion, which may cause up to 60% of nitrogen release within 48 h [86,87]. Multilayer strategies, which utilize layers of alternating anionic alginate and cationic polymer layers (chitosan, poly-L-lysine) via layer-by-layer (LbL) assembly, avoid this shortcoming, with each bilayer adding 5–15 µm of thickness and decreasing porosity. A three-bilayer alginate–chitosan coating extended nitrogen release by a factor of 3, to over 25 days, whilst retaining a cumulative release of 85% over 30 days [88]. Core–shell designs with the combination of inner encapsulation of alginate and outer densely crosslinked shells coordinate the availability of nitrogen to crops with phenological demand. Nano-additives such as montmorillonite nanoclay, nano-ZnO and graphene oxide sheets in coating layers form tortuous diffusion routes, which decrease first-day burst release by 40–55% [89]. The microalgae–chitosan–starch composite complex network is a promising example, with a urea encapsulation capability of 440%, and CNF-alginate-PVA hydrogels have abilities to retain 80% moisture for 60 days with customized delivery of nutrients in soil [90]. The nutrient slow-release kinetics are non-Fickian Higuchi from the transitional state of swelling to steady-state diffusion and coating matrix biodegradation in 10 to 30 days. The transport mechanism (Fickian vs. anomalous) can be distinguished using SAXS (mesh size ξ governing diffusion regime) and DSC (bound water fraction), as discussed in Table 2. The salt-sensitive alginate recipes maintain functional characteristics in NaHCO_3_/Na_2_CO_3_ media with effective buffering of Na^+^ in the rhizosphere. Saline–alkaline soils experience micronutrient deficiency, especially when higher pH levels leach off Fe, Zn, Mn and Cu into unavailable states. Calcium alginate resolves this by complexing chelated metal, i.e., Fe-EDTA, Zn-EDTA and Fe-EDDHA, within the gel, preventing their precipitation but facilitating pH-controlled release via the acidification of the rhizosphere (Figure 5) [91]. ZnSO_4_-loaded alginate beads can support Zn^2+^ release in 20 to 35 days in calcareous soils [92]. Fe-loaded alginate chitosan microspheres can be used to overcome iron (Fe) deficiency chlorosis on alkaline substrates. Encapsulation of boron can be used to eliminate phytotoxic spikes whilst maintaining a steady-state level of boron in the optimal range. Traditional slow-release fertilizers with polymer coatings release nutrients gradually, prolonging the release time but leaving persistent microplastic traces. Biodegradable alginate-based formulations, including but not limited to cellulose–starch–alginate, with better biomass and soil porosity without microplastics require further improvement in the context of mechanical strength in wet–dry cycles [93,94]. The composites of alginate-based materials and outer coatings of modified natural waxes, or zein protein, have enhanced durability and biodegradability. This reveals a glimpse of the next generation of coated urea products that can improve saline–alkaline soils [95,96].

## 6. Alginate-Coated Nucleic Acid Delivery System to Plants

Alginate-based hydrogels are a macromolecular platform for the encapsulation and effective delivery of various types of nucleic acid cargoes, as discussed in Table 3A and B, e.g., CRISPR-Cas9 ribonucleoprotein (RNP) complex, messenger RNA (mRNA), long non-coding RNA (lncRNA), small interfering RNA (siRNA) and RNA interference (RNAi), to plant tissues [97,98]. The anionic polysaccharide backbone of alginate comprises mannuronic (M) and guluronic (G) acid residues, which can be used for crosslinking to form gels that protect genetic material from nuclease-based degradation and controlled release in response to a stimulus. High guluronic acid formulations maintain structural integrity over a pH range of 5.0–10.5 and electrical conductivity of over 10 dS m^−1^, indicating their suitability for a salt–alkaline regime [99,100]. Encapsulation efficiencies greater than 99% with salt-responsive buffering have been achieved by electrospinning and electrospray techniques. Nucleic acid delivery into plant cells is more challenging than in mammalian systems due to the stiffness of the cellulose hemicellulose pectin cell wall, which has a diameter of 5–20 nm. The cellular uptake or release repulsion of negatively charged nucleic acid is due to the presence of negative charges in the plasma membrane [101,102]. Conventional methods, i.e., agrobacterium-mediated transformation, particle bombardment and polyethylene glycol (PEG)-based transfection of the protoplast, are species-specific, prone to chromosomal integration, damage the cells and are not readily scalable to field level, especially under variable environments [103]. The calcium alginate-based preparations address the following challenges via the production of tunable sub-20 nm nanobioparticles and combined cationic crosslinkers, i.e., chitosan, poly-L-lysine and polyethyleneimine [104]. These coupling crosslinkers condense nucleic acids into positively charged nanocomplexes entrapped in the anionic alginate matrix, with favorable ionic gelation or surface coating through different layered assemblies [105]. The resulting core–shell structures of the biocompatible alginate shell and cationic core shield the nucleic acid cargo from extracellular nucleases, pH-induced hydrolysis, ionic degradation in rhizospheres and stimuli release in response to pH, ionic strength or microbial enzymes [106]. These nanocomplexes can be applied to part or the whole plant using different methods, i.e., rhizosphere release for root-specific genes via root drenching, stomatal uptake for leaf via foliar spraying, gene activation for vulnerable early germination stages and enhanced tissue penetration resulting from vacuum infiltration methods [107]. All these methods lead to established agronomic growth without requiring tissue culture, sterile facilities and gene-dependent protocols that minimize the barriers for resource-meager environments [108].

## 7. Delivery of CRISPR-Cas9 Using Alginate-Encapsulated Nanoparticles

**Figure 6:**
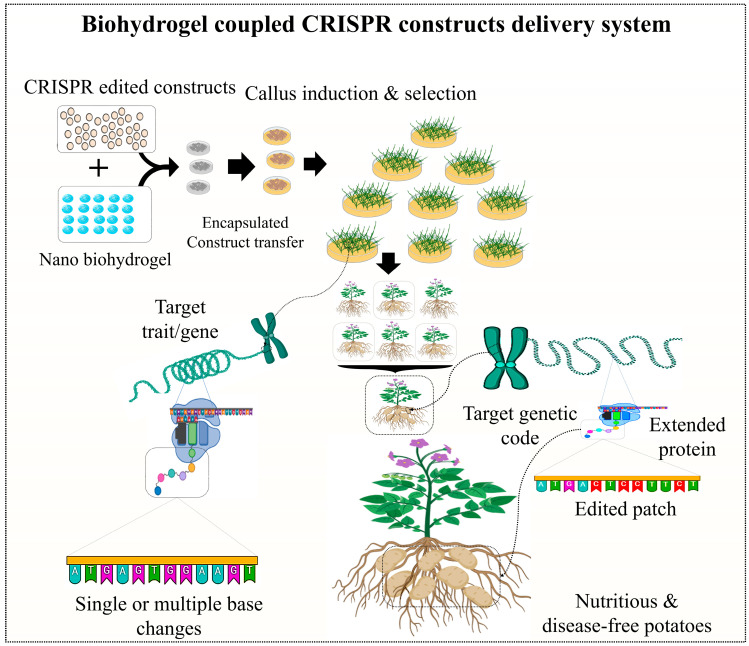
Nanobiohydrogel-mediated CRISPR construct delivery system. Transfer of CRISPR constructs encapsulated in nanobiohydrogels into plant cells followed by callus induction, selection and regeneration of edited modification to produce healthy and disease-resistant varieties of potatoes. The image is adopted from [139] and modified under the terms of the Creative Commons Attribution License (CC BY).

The encapsulation of CRISPR-Cas9 ribonucleoproteins (RNPs) in calcium alginate hydrogels offers the possibility to edit the genes responsible for salt tolerance without integrating into the genome, as shown in Table 3A [132]. This approach eliminates the risk of insertional mutagenesis and avoids GMO regulatory status in many countries, thereby supporting agricultural adoption and cooperation [132]. The preassembled Cas9-sgRNA complex is inherently temporary and performs the desired edit before being destroyed by host proteins and nucleases, leaving no foreign DNA traces in the host genome (Figure 6) [133]. The RNP complex (~160 kDa, ~10 nm diameter) has a net negative charge in physiological pH, requiring cationic condensation with chitosan or PEI at optimal N/P ratios to produce 30 to 80 nm nanocomplexes that are then embedded within the alginate and crosslinked with Ca^2+^ to yield a core–shell nano counterpart with a 90% encapsulation rate [134,135]. The ionic gelation conditions, ambient temperature, aqueous phase and neutral pH are sufficient to preserve for the folding and structural stability of the Cas9-sgRNA complex, as compared to harsh organic solvents and sonication conditions of lipid nanoparticle systems during coating. The main genic players for salt tolerance are *HKT1 (Na^+^ retrieval from xylem), SOS1 (Na^+^ extrusion from root cells), and AVP1 (vacuolar Na^+^ sequestration via a tonoplast proton pump),* which increase the sequestration of Na^+^ in vacuoles [136]. The cis-regulatory elements can be edited to activate these genes without changing coding sequences, and negative regulators like *GmNAC06* or *OsRR22* can be knocked out to de-repress endogenous defense [113,137]. Editing efficiencies of 80–99% in *Triticum aestivum*, *Oryza sativa*, and *Zea mays* have been achieved using alginate-protected coatings with optimum on-target specificity in the rhizosphere of pH 6.5–8.5 and other standard conditions [135]. The salt-responsive release of alginate RNP achieves DNA-free edits in the T0 breeding cycles, reducing the breeding window by two–four generations. Critical gaps exist for the efficiency of edits in assessing multi-generational genotype trait stability, microbial conditions and setting biosafety standards to deal with off-target editing and ecological consequences [111,138].

## 8. Alginate-Coated Salt Stress-Responsive mRNA and lncRNA Delivery

**Figure 7:**
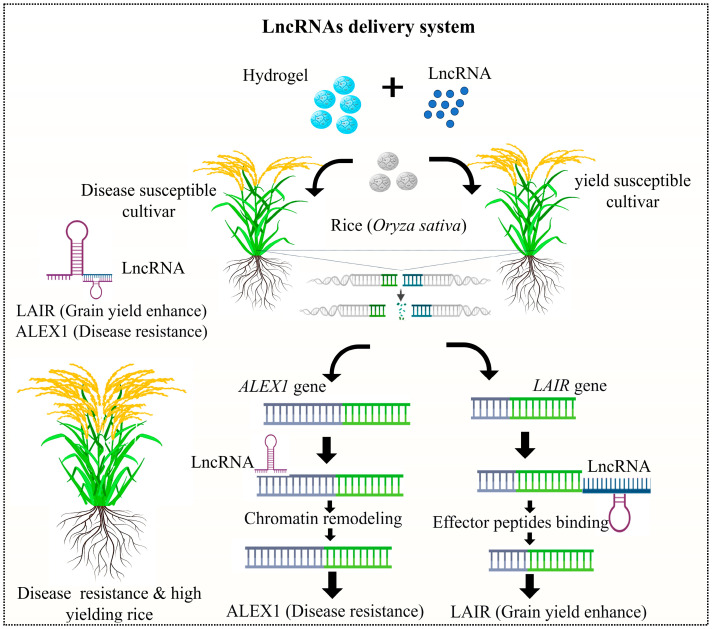
Hydrogel long non-coding RNA (LncRNA) delivery system. LncRNAs loaded in hydrogel carriers regulate *ALEX1* and *LAIR* genes via chromatin remodeling and disease resistance and grain yield in rice.

Alginate–cellulose composite-encapsulated messenger RNA and long non-coding RNA (lncRNA) enable transient expression of stress-protective proteins without permanent changes in DNA, as shown in Figure 7 and Table 3B [140]. The foreign mRNA does not enter the nucleus but is translated by the ribosomes within a few hours of being delivered to the cell, and the alginate coating extends the expression window from 24 to 72 h. The coating extends the expression window by acting as a sustained-release reservoir that replenishes cytoplasmic mRNA pools. The important targets are enzymes to produce osmolyte, i.e., trehalose-6-phosphate synthase (TPS), to regulate proline content, i.e., proline dehydrogenase (ProDH), to protect from dehydration LEA proteins (late embryogenesis abundant) and to scavenge ROS superoxide dismutase (SOD). LncRNA-coated hydrogels for *ALEX1* and *LAIR* genes lead to disease and grain yield improvements in rice [141,142]. The transient nature of expression is a key advantage to produce salt tolerance proteins in the required time to avoid the high energy costs of constitutive expressions of transgenes. Alginate-coupled cellulose encapsulates shield cargo from extracellular RNase degradation, alkaline damages (pH > 8.0) and metal ion-induced degradation under greenhouse conditions for 60 days [131]. Structured lncRNAs are slightly more nuclease resistant than RNase-prone mRNA, while the functional half-life of both is increased by alginate encapsulation. The chemical modifications, e.g., pseudouridine, 5′ capping and poly(A) tail, also enhance the translational yield of these transgenes. Long non-coding RNA (lncRNA) serves as regulatory molecules, controlling gene expression at transcriptional, post-transcriptional and epigenetic levels [112,143]. Epigenetic modification-based lncRNA functions in ion control by recruiting chromatin remodeling complexes to Na^+^/K^+^ transporters, and promoters regulate the alternative splicing of stress-responsive genes. In this mechanism, LncRNA acts as competing endogenous RNA and sequesters microRNAs to stop the function of salt tolerance-related mRNA targets in the crops. Alginate-coated lncRNA systems are promising in terms of antioxidative activity and ion transport regulation, but field performance is currently unknown due to the challenges of larger size and encapsulation conditions [144]. Their larger size reduces loading and release efficiency compared to mRNA; however, alginate porosity can be tuned to compensate for this limitation. Both cargoes are for transient expressions that integrate freely and are favorable for biosafety, yet they exhibit dose-dependent response variability and need multiple applications. The effect of mRNA on osmolyte and ion balance has been more clearly observed in plants, while the effect of lncRNA is more regulatory and not yet validated at the field scale. Major challenges are the low ionic strength of mRNA stability, lack of synchrony for osmolyte expression with the duration of stress perturbations and need for detailed dose–response studies on plants and plant growth stages [145]. The scale up of these RNA–alginate systems has not been well tested in the field beyond the laboratory and greenhouse. There are several further limits to deployment, including the cost of GMP-grade RNA and the scalability of alginate fabrication. The regulatory landscape for RNA-based crop interventions is also emerging as a new regulatory pathway apart from the transgenic GMO regulatory pathway [146,147].

## 9. Salt-Responsive siRNA and RNAi Delivery for Silencing Salt-Sensitive Genes

**Figure 8:**
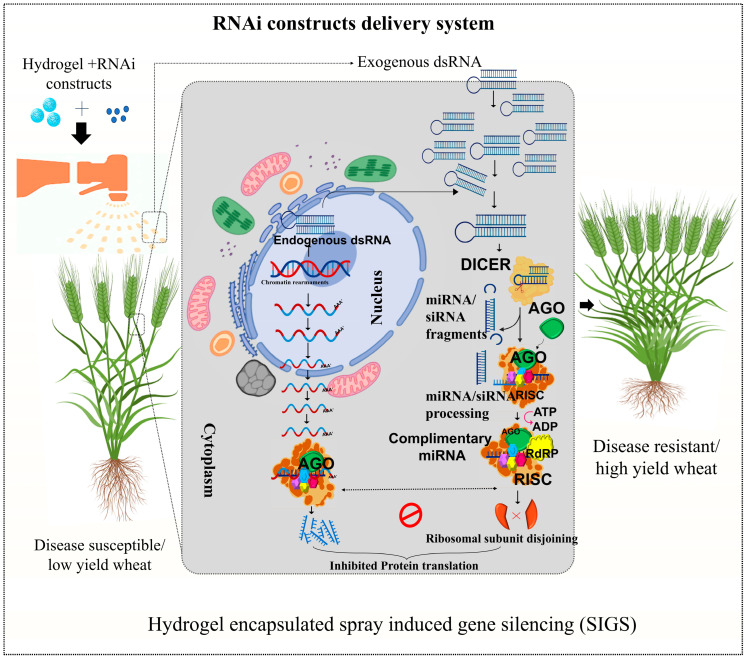
Hydrogel-based spray-induced gene silencing (SIGS) for disease-resistant and high-yielding wheat varieties. The RNAi-based technology allows rearrangements of chromatin and post-transcriptional silencing of genes on the basis of susceptibility, which is a non-transgenic method of crop improvement. The image is adopted from [139] and modified under the terms of the Creative Commons Attribution License (CC BY).

Alginate-coated small interfering RNA (siRNA) and RNA interference (RNAi) molecules deliver sequence-targeted suppression of negative regulators of salt tolerance without the need for introducing foreign protein-encoding sequences, given in Figure 8 and discussed in Table 3B [115]. The RNAi-mediated processing of the double-stranded RNA (dsRNA) precursors by Dicer-like enzymes generates 21 to 24 nucleotide siRNA duplexes. The formed complexes guide RISC (RNA-induced silencing complex)-mediated degradation of complementary mRNA targets, which allows for the specific silencing of genes involved in antagonizing salt tolerance [148]. The core target and main gene is *STOP1* (sensitive to proton rhizotoxicity1), whose continuous expression in conditions of high alkalinity diverts plant resources from salt adaptation, especially *NCLX* (Na^+^/Ca^2+^ exchanger). This exacerbates the low cytosolic Ca^2+^ concentrations in the presence of high Na+ ions, *OsHKT2;1* mediated excessive Na^+^ root influx and negative regulators of the SOS signaling pathway [7]. The perfect sequence complementarity of hydrogel-coated siRNA targeting STOP1 reduces pleiotropic off-target effects, giving siRNA an edge over chemical broad-spectrum suppressors. Uncoated dsRNA lasts only hours in saline rhizospheres due to ribonuclease (RNase) degradation, alkaline degradation and adsorption on soil particles [149]. Alginate coating extends the half-life from hours to days or weeks, providing nuclease protection and pH buffering. The salinity-dependent swelling of alginates allows the release to be tailored to salinity, and increased siRNA doses are released at times of stress. In fact, alginate encapsulation has been demonstrated to have 60–80% silencing efficiency compared to the rapid degradation of conventional dsRNA under similar conditions [126,150]. The combination of alginate with spray-induced gene silencing (SIGS) makes the technology feasible for field applications [147]. Alginate–chitosan nanocomplexes were designed as sprayable suspensions with uniform coating, rain fastness of 48 h, and a slow-release duration of 7–14 days [151]. Research challenges persist, particularly in the optimization of coating gradients, determining the right ionic strength on the selectivity of off-target silencing, duration of the phenotype, ecological consequences of beneficial rhizosphere microbiome and multiplexing of formulations in targeting other negative regulators [130,152].

## 10. Conclusions and Future Perspectives

Calcium alginate-based hydrogel formulations are a convergent polymer platform for combined encapsulation of nutrients and delivery of nucleic acids for the alleviation of saline and alkaline stress. The egg-box supramolecular crosslinked structure allows for the controlled porosity, encapsulation, stress-triggered release, shielding of nutrients and nucleic acid functional constructs from pH-catalyzed hydrolysis, ionic and nuclease degradation. The hydrogel-coated CRISPR-Cas9 ribonucleoprotein complexes targeting salt tolerance genes, e.g., *HKT1*, *SOS1* and *AVP1,* accomplished promising outcomes and reduced generational breeding windows of two–four growth seasons under controlled conditions. The genetically specific silencing of the negative traits of salt regulatory genes and transient expression of osmolytes by hydrogel entrapped with siRNA and mRNA increased the yield in cash crops. Several studies demonstrated outcomes by using alginate hydrogel-coated nutrient delivery, which aided in reducing nutrient leaching, promising water use efficiency and delayed water demand with improved yield. Key challenges that hinder field-scale translation remain to be addressed, including mechanical durability under prolonged wet–dry cycles, encapsulation stability, and the timing of cargo release. The phenological demand of crops and ionic strength influence the encapsulation stability and specific off-target genome editing in different genotypes and are still elusive, requiring rigorous investigation to establish the pivotal role of hydrogel-based amendments. Importantly, the lack of life cycle analyses, biosafety frameworks of coatings and multi-season agronomic cost–benefit analysis are key barriers to accurate yield estimation and production. Multi-site and multi-season field validations are urgently needed under heterogeneous edaphic conditions to translate calcium alginate biohydrogels from controlled environments to scalable industrial biomaterial applications. This approach can help restore the productivity of 1.5 billion ha of salt-affected lands. Future research should focus on improving the ionic stability and sustainability of bio-compatible hydrogels to enhance their performance. Artificial intelligence-based inverse formulation design for encapsulation, multiplexed co-delivery systems for nutrients and genetic complexes with sensor-integrated decision support systems under real-time frameworks are required. Computer vision and artificial intelligence-based real-time information processing systems are needed to meet the demands of food security and sustainable agriculture.

## Figures and Tables

**Table 1:** Frameworks for molecular characterization of alginate-based hydrogel-coated formulations.

Method/Technique	Scale	Parameters	Ions (Na^+^/Ca^2+^/Mg^2+^) Exchange	Nutrient Encapsulation and Behavior	Ref.
Solution NMR (^1^H, ^13^C)	0.1–1 nm	M/G ratio NG > 1, NM > 1 epimer distribution	High-G blocks form denser egg-box, Na^+^ shift predictor	M/G-dependent carboxylate density controls NH_4_^+^, K^+^, & NO_3_^−^ affinity	[12,36,39,47,48]
NMR (^23^Na MAS-NMR), ^13^CCP/MAS	0.1–1 nm	Chemical shift (ppm), Cq, direction symmetry	Ca^2+^ egg-box coordination from disordered Na^+^ binding in situ	Quantifies divalent cation binding site occupation for Zn^2+^ & Mg^2+^	[39,47,49]
GPC/SEC (with RI + MALS)	1–100 nm (Chain)	Mw, Mn, PDI, Rg (nm), intrinsic viscosity (η)	High Mw alginate networks resisting Na^+^ in saline soil	Mw → swelling ratio & diffusion path length for N-species release	[12,50]
FTIR/ATR-FTIR	0.1–1 nm	Δν split, COO^−^ peak position (~1600/~1420cm^−1^), crosslinking index	Carboxylate band shifts quantify Ca^2+^ to Na^+^ detachment & structural loosening	Identifies NH_4_^+^, urea & phosphate encapsulation	[12,39,48,49]
Raman Spectroscopy (Confocal mapping)	~0.1–10 nm, (~0.5 µm)	Cargo fingerprint bands, Peak positions (carboxylate/OH/CH), 2D intensity maps	Ca^2+^ bands shift detection upon Na^+^ loosening	Maps distribution of nutrient (urea, NH_4_^+^) in bead cross sections	[49,51]

**Table 2:** Frameworks for alginate hydrogel based encapsulates characterization.

Analytical Class	Technique	Scale	Parameters	Ions (Na^+^/Ca^2+^/Mg^2+^) Exchange	Nutrient Encapsulation and Release	Ref.
Chemical form	XPS	1–10 nm (lateral) 10–200 µm	Ca 2p, Na 1s, O1s, N1s (eV), Ca/Na atomic (%)	Quantifies surface Ca^2+^loss, & Na^+^ gain, depth profiling	Tracks Fe^2+^/^3+^, Zn^2+^ & NH_4_^+^ surface binding for fertilizer granules	[39,52,53,54]
Conformational assembly	XRD/Wide angle X-ray diffraction	0.1–10 nm	D-spacing (~4.4 Å), peak FWHM, Coherence length, crystallinity index (CI)	CI decreases as Na^+^ replaces Ca^2+^, structural distortion in saline stress	Higher CI = greater matrix rigidity = slow release of NH_4_^+^, NO_3_^−^, & PO_4_^3−^	[12,36,39,49]
Secondary structure	Circular dichroism (CD) spectroscopy	1–10 nm	Molar ellipticity (θ) Cotton effect amplitude, transition midpoint	Helical order loss, Ca^2+^ to Na^+^ loosening in gel network	Structural relaxation under ionic stress directly correlates with N-species release rate	[7,39,51,55]
Network morphology	SEM, Cryo-SEM reach ~10 nm in FEG mode.	10 nm–100 µm	Diameter, structure breadth, porosity, surface roughness	Visualizes pore collapse or swelling, surface erosion in NaCl	Pore architecture → N retention vs. leaching, open pores → burst, closed → slow release	[4,12,56]
Nanostructures	TEM/ Cryo-TEM	0.5–100 nm	Junction zone width, NPs diameter, Thickness, core density	Directly visualizes junction zone disrupted by Na^+^, hidden fixes of SEM	Confirms nutrient encapsulated vs. surface-adsorbed	[8,12]
Behavioral analogy	FRAP (Fluorescence recovery after photobleaching)	1–100 nm (Mol.), 1–100 µm (Bleached)	D, t_½_ mobile fraction (%), (D/D_0_)	Real-time ions mobility in saline stress deprived of gel disruption	Spatiotemporal controlled diffusion & immobilized cargoes	[39]
3D architecture	CLSM (Confocal laser scanning microscopy)	0.5–100 µm	3D cargo locality, diffusion front velocity, mobile fraction (%)	Real-time structural imaging polymer formulations	Visualizes spatio-temporal nutrient diffusion gradients & localization	[15]
Polymer network architecture	SAXS/SANS	1–100 nm	Lp, ξ, fractal dimension (D)	Quantifies real-time ξ change in salt	ξ directly governs NH_4_^+^, NO_3_^−^, H_2_PO_4_^−^ diffusion, Fickian release dose	[39,40]
Mechanical properties	Oscillatory rheology (G′, G″, tan δ)	Bulk (mm–cm)	G′ (Pa), G″ (Pa), tan δ, tgel, crossover frequency	Real time G′ decline tracks Ca^2+^ → Na^+^ exchange, G′ < 100 Pa signals gel failure	G′ determines release duration: soft gels (100–500 Pa) & (>2000 Pa) so on.	[39,40,55,57]
Mechanical properties	Compression/tensile testing	Bulk (mm–cm)	E (kPa-MPa), strain failure (%), mJ, strength (kPa)	Quantifies bead strength in Ca^2+^→Na^+^ exchange, field integrity	Mechanical toughness ensures fertilizer beads survive tillage, & irrigation before release	[40,58]
Mesh size	Swelling studies (Flory-rehner model)	1–100 nm	Q, EWC (%), ρx (mol/m^3^), ξ	ΔQ under NaCl gradient quantifies osmotic imbalance	Q predicts diffusion-controlled N release rate, high Q = faster delivery to rhizosphere	[4,56,57]
Movement behavior	PFG-NMR diffusion (Pulsed field gradient)	1–100 nm	D (m^2^/s), D/D_0_, τ	Real time Na^+^/Ca^2+^ kinetics measured in situ under NaCl	Provides NH_4_^+^ mobility, & tortuosity (τ), essential inputs for release models	[39,59,60]
Movement behavior	In Vitro Release (Franz cell/dialysis)	Bulk (mm–cm)	Cumulative release (%), t_50_(h), Korsmeyer-Peppas (n), k, burst fraction	Simulates saline field conditions, measures Na^+^ induced accelerated release kinetics	Gold standard for slow-release fertilizer characterization, regulates Fickian vs. anomalous mechanism	[4,33,50,56,57]
Movement behavior	Ion selective electrodes	Bulk (solution)	C (mM), exchange rate (µmol/g/h), Kd	Monitoring of Ca^2+^ efflux, & Na^+^ influx during saline challenge	High-resolution NH_4_^+^/NO_3_^−^ release curves, enables direct slow-release vs. conventional contrast	[50]
Pore architecture	Micro-CT/ X-ray tomography	1 µm–1 mm	Porosity (%), pore volume (cm^3^/g), connection, spherical Index, τ	Visualizes channel collapse under ionic imbalance, simulates salt/water transport	Distinguishes macropore burst release vs. diffusion controlled sustained N release	[4,61]
Pore architecture	BET surface area (N_2_ adsorption)	1–300 nm	SBET (m^2^/g), BJH pore distribution (nm), Vp (cm^3^/g)	Higher SBET → <carboxylate sites, accelerating Ca^2+^ → Na^+^ exchange rate	SBET determines NH_4_^+^, K^+^, Zn^2+^, Fe^3+^ loading capacity per gram of carrier	[58]
Image based porosity	SEM/CLSM image Analysis (FIJI/ImageJ 1.54f with TWS plugin v3.3.4)	50 nm/100 µm	Mean pore diameter (µm), pore area fraction (%), circularity, strut width (µm)	Quantifies pore size change	Provides pore geometry for effective medium diffusion models parameterizing N release	[4]
Surface chemistry	DLS (Dynamic light scattering)	1–1000 nm	Z-average Dh (nm), PDI, DT, size distribution	Detects nanoparticle in Na^+^/Mg^2+^ charge screening in saline medium	Optimizes nanoparticle size, & colloidal stability in irrigation water	[62]
Surface chemistry	NTA (Nanoparticle tracking analysis)	10–1000 nm	Dh, modal diameter (nm), concentration (particles/mL)	Intensity deconvolution	Particle concentration accurate dose of nutrient	[63,64]
Surface chemistry	Zeta Potential (Laser doppler)	1–1000 nm	ζ (mV), pI, |ζ| > 30 mV	Na^+^ compress double layer, reducing (ζ) to combination levels	Negative ζ governs NH_4_^+^, K^+^, Fe^3+^ binding, nutrient loading charge	[64,65]
Surface chemistry	AFM+ Nanoindentation	1–100 nm	Ra/Rq (nm), E (kPa), adhesion force (nN), peak force maps	Force distance curves quantify surface softening, & stiffness	Force spectroscopy detects nutrient binding at particle level	[39,50,51,55]
Surface chemistry	Fluorescence spectroscopy /FRET	1–100 FRET: 1–10 (nm)	FRET efficiency E (%), donor-acceptor distance r (nm), EE (%), intensity (a.u.)	FRET probes report Ca^2+^→Na^+^ cause conformational change in gel	Tracks labelled nutrient analog release with nM sensitivity, enables droplet measurements	[66]
Interface chemistry	ToF-SIMS	1–10 nm, ~1 µm	Ca^+^/Na^+^ ratio maps, molecular ions	Maps Ca^2+^ → Na^+^ replacement gradient from surface to core	Distinguishes embedded vs. surfaced Fe^2+^, Zn^2+^, NH_4_^+^ profiling	[39,53,54]
Interface chemistry	QCM-D	1–100 nm (films)	Δf (Hz → ng/cm^2^), ΔD, hydrated film mass, rigidity ratio	Nanogram tracking of Ca^2+^ crosslinking & Na^+^ decrosslinking	Calculates nutrient adsorption & Kd on alginate coatings	[67]
Interface chemistry	EDS/EDX (SEM-EDS/STEM-EDX)	100 nm to 10 µm	Elemental wt%/at%, Ca/Na ratio gradient across beads	Maps Ca^2+^ → Na^+^ exchange gradient at µm spatial resolution	Confirms nutrient encapsulation depth, localizes N, P, K, Zn, Fe distribution beads	[12,54]
Thermal property elucidation	DSC (Differential scanning calorimetry)	Bulk/mg scale	ΔH (J/g), wt%, Tg/Tm (°C), crosslinking density	Increased free water fraction upon Na^+^ release of Ca^2+^ signals crosslink disruption	Absorbed water fraction correlates → nutrient retention, high bound water = slower release	[39,55,56,58]
Ionic quantification	ICP-OES/ICP-MS	Bulk (µg/L–mg/L)	Concentration (mg/L), Ca^2+^ content (mmol/g), exchange capacity (meq/g), selectivity coefficient	Gold standard for quantifying Ca^2+^ loss, & Na^+^/Mg^2+^ gain, validate selectivity coefficient	Precisely quantifies N, P, K, Fe, Zn loading, & cumulative release, enables NUE calculations	[50]

**Table 3:** A. Multiscale alginate hydrogels encapsulated with CRISPR-Cas9/cargo bearing plasmid delivery systems. B. Alginate hydrogels protected mRNA LncRNA, siRNA/RNAi/dsRNA and nutrient-coated delivery systems.

Carrier	Fabrication	Encapsulate	Function	Delivery Route	Outcomes	Ref.
A						
Ca-alginate/chitosan NPs (30–80 nm)	Chitosan/PEI condensation, crosslinking, Ionic gelation, N/P 10:1, Ca^2+^	CRISPR-Cas9 RNP (~160 kDa, ~10 nm)	*HKT1* (Na^+^ retrieval) *SOS1*, (Na^+^ extrusion) *AVP1*(vacuolar pump)	Root drench, pH triggered release	EE > 90%, 80–99% editing, generational time compression, Salt-responsive	[109,110,111]
Alginate-PEI electrospray nps (228 nm)	Electrospray, alginate/CaCl_2_, PEI condensation	Dual CRISPR pDNA	*GmNAC06* or *OsRR22*	Vacuum infiltration, protoplast transfection	EE > 99%, pDNA intact (FTIR), DSB confirmed, cytocompatibility, pH 5–10.5 stable	[104,112,113]
MSN alginate composite (<20 nm)	MSN functionalization, PEI/PLL coating	Cas9 pDNA-coated mnps + sgRNA, siRNA multi-gene	*OsERF922* (blast resistance) *SD1* (semi-dwarf, *Wx* (glutinous starch)	Root soak 48 h, foliar spray, fruit injection	Cortex + endoderm penetration, vascular transport, nuclear delivery, long-term silencing	[114,115]
DNA-coated MNPs (Fe3O4)	Fe_3_O_4_ + DNA adsorption, magnetofection	pDNA (CRISPR construct)	Grain quality, yield Herbicide resistance, waxy starch (*GBSS1*)	Pollen magnetofection, field-scale	Genotype independent Maize bypasses tissue culture, germline delivery, 400% efficiency gain	[116,117]
Chitosan-PLGA nps (160 nm)	Chitosan coating PLGA, Double emulsion	Cas9-sgrna pDNA	salt tolerance regulators, Cis-regulatory upregulation	Foliar spray, Seed coating	80% DNA EE, burst ~24 h, pH-responsive, low cytotoxicity, biodegradable	[118]
B						
mRNA Delivery (Transient Expression)						
Alginate-cellulose composite microspheres	Ionic gelation, CMC blending, 5′ cap + pseudouridine	mRNA (*TPS*, *ProDH*, *LEA*, *SOD*)	Trehalose synthase, proline pools, *LEA* desiccation, ROS scavenging	Foliar spray, root drench, sustained 24–72 h	Transient expression, no genetic integration, stable > 60 d, protected pH > 8.0, no constitutive burden	[119]
Ca-alginate/PLL nanohydrogel (<20 nm)	PLL condensation, Ionic gelation	Modified mRNA polyA optimized	*NHX1* antiporter, *PIP* aquaporins, osmolyte enzymes	Seed priming, germination activation	Bypasses nucleus, ribosomal Translation, sustained mRNA pool, salt-alkali compatible	[120,121]
LncRNA Delivery (Epigenetic Regulation)						
Alginate-chitosan IPEC	Electrostatic IPEC Chitosan/alginate crosslink	LncRNA (Salt responsive)	Na^+^/K^+^ transporter promoter scaffolding, miRNA, Chromatin remodeling	Foliar spray, root zone delivery	Chromatin modulation at ion transporter loci, antioxidant efficiency improved, size optimization needed	[122,123]
siRNA/RNAi/dsRNA (gene silencing)						
Alginate-chitosan dsRNA nps	Chitosan/dsRNA, alginate complexation, electrostatic assembly	dsRNA (long + 21–24 nt siRNA)	*Mopmk1* (Rice blast), *STOP1, OsHKT2;1* (Na^+^ influx)	SIGS foliar spray rain fast > 48 h, 7–14 d release	Sequence specific + stress dual activity, reduced blast disease, field-scale SIGS, biodegradable carrier	[124]
Guanidinium-siRNA nps	Guanidinium polymer/ siRNA complexing, cationic condensation	SiRNA (21nt) delivery	Long distance systemic silencing, inter-organ & species	Foliar spray, phloem mediated translocation	Long distance knockdown, inter organ silencing, high uptake efficiency	[125,126]
Ca-alginate salt-stable matrix beads	CaCl_2_ crosslinking	siRNA (21–24 nt, RISC-loaded)	*NCLX* Na^+^/Ca^2+^ exchanger, SOS cascade negative regulators, salt-sensitive suppressors	Root drench, salinity- proportional dose	Nuclease barrier + pH buffer, bioavailability weeks vs. hours, 60–80% silencing, Salinity proportional release	[115,127]
EV-mimetic nanovesicles	dsRNA loading, Spray formulation, Membrane chimeric	dsRNA, SIGS-optimized	pathogen virulence genes, TMV/fungal targets, pest resistance	SIGS spray, uniform coverage, 20 d protection	97.2% silencing (24 h), EV mimetic enhanced CME uptake, rain fast, field-compatible, biodegradable	[127]
Multi-Cargo Co-Delivery (Genetic + Nutritional)						
Alginate-CNF-PVA hybrid	Composite nanocellulose, dual encapsulation, co-release design	siRNA + NPK (Co-delivery)	Salt negative regulators + nutritional supplementation	Soil amendment, seed coating	Simultaneous genetic + nutritional, 30–40% reduced N-leaching, moisture > 80% (60 d), no microplastic	[128]
LDH-alginate nanosheet composite	LDH-dsRNA layering, Alginate coating	DsRNA + micronutrients (Zn, Fe)	TMV, TYLCV viral genomes, pathogen virulence, nutrients	Foliar spray, soil drench, pH responsive	dsRNA stable pH 11, >20 d protection, 90% pest mortality vs. naked dsRNA	[129,130,131]

## Data Availability

No new data were generated or analyzed in this review article.

## Abbreviations

A list of abbreviations used in the main text is given in the list of abbreviations:

**Abbreviations**	**Details**	**Abbreviations**	**Details**
*HKT1*	High-affinity K^+^ Transporter 1	*TPS*	Trehalose-6-phosphate synthase
*SOS1*	Salt overly sensitive 1	*ProDH*	Proline dehydrogenase
*AVP1*	Arabidopsis vacuolar h^+^-pyrophosphatase 1	*LEA*	Late embryogenesis abundant
*NHX*	Na^+^/H^+^ antiporter	*SOD*	Superoxide dismutase
*NCLX*	Na^+^/Ca^2+^ exchanger	*CAT*	Catalase
*STOP1*	Sensitive to proton rhizotoxicity 1	*PIP*	Plasma membrane intrinsic protein
*OsHKT2;1*	*Oryza sativa* high-affinity K^+^ transporter 2;1	*GBSS1*	Granule bound starch synthase 1
*GmNAC06*	*Glycine max* NAC transcription factor 06	*SD1*	Semi-dwarf 1
*OsRR22*	*Oryza sativa* response regulator 22	*Wx*	Waxy gene
*ALEX1*	Awakening of lineage-specific expression 1 (LncRNA)	*LAIR*	Leucine-rich repeat receptor-like kinase associated LncRNA in rice
CRISPR	Clustered regularly interspaced short palindromic repeats	PVA	Polyvinyl alcohol
Cas9	CRISPR-associated protein 9	PEI	Polyethyleneimine
RNP	Ribonucleoprotein	PLL	Poly-L-Lysine
sgRNA	Single guide RNA	PLGA	Poly (lactic-co-glycolic acid)
pDNA	Plasmid DNA	LDH	Layered double hydroxide
mRNA	Messenger RNA	LbL	Layer by layer
lncRNA	Long non-coding RNA	DSB	Double strand break
siRNA	Small interfering RNA	GMO	Genetically modified organism
dsRNA	Double-stranded RNA	ROS	Reactive oxygen species
miRNA	MicroRNA	MDA	Malondialdehyde
RNAi	RNA interference	PSII	Photosystem II
RISC	RNA induced silencing complex	Fv/Fm	Maximum quantum efficiency of PSII
SIGS	Spray induced gene silencing	G	Guluronic acid (α-L-guluronic acid)
WUE	Water use efficiency	M:G ratio	Mannuronic to Guluronic acid ratio
NUE	Nutrient use efficiency	CMC	Carboxymethyl cellulose
H_2_O_2_	Hydrogen peroxide	CNF	Cellulose nanofiber
M	Mannuronic acid (β-D-mannuronic acid)	CP/MAS	Cross polarization magic angle spinning
LCST	Lower critical solution temperature	PFG-NMR	Pulsed field gradient NMR
IPEC	Interpolyelectrolyte complex	GPC	Gel permeation chromatography
PEG	Polyethylene glycol	SEC	Size exclusion chromatography
MSN	Mesoporous silica nanoparticle	MALS	Multi angle light scattering
MNPs	Magnetic nanoparticles	RI	Refractive Index
NPs	Nanoparticles	FTIR	Fourier transform infrared spectroscopy
EV	Extracellular vesicle	ATR-FTIR	Attenuated total reflectance FTIR
NMR	Nuclear magnetic resonance	XRD	X-ray Diffraction
MAS-NMR	Magic angle spinning NMR	XPS	X-ray photoelectron spectroscopy
TEM	Transmission electron microscopy	CD	Circular dichroism
Cryo-SEM/TEM	Cryogenic SEM/TEM	SEM	Scanning electron microscopy
FEG	Field emission gun	SANS	Small angle neutron scattering
CLSM	Confocal laser scanning microscopy	Micro-CT	Micro computed tomography
FRAP	Fluorescence recovery after photobleaching	BET	Brunauer-Emmett-Teller (surface area analysis)
SAXS	Small angle X-ray scattering	BJH	Barrett-Joyner-Halenda (pore distribution)
DLS	Dynamic light scattering	ICP-MS	Inductively coupled plasma mass spectrometry
NTA	Nanoparticle tracking analysis	Mw	Weight-average molecular weight
AFM	Atomic force microscopy	Mn	Number average molecular weight
FRET	Förster resonance energy transfer	PDI	Polydispersity index
ToF-SIMS	Time of flight secondary ion mass spectrometry	Rg	Radius of gyration
QCM-D	Quartz crystal microbalance with dissipation	Lp	Persistence length
EDS/EDX	Energy dispersive x-ray spectroscopy	ξ	Mesh size
STEM	Scanning transmission electron microscopy	CI	Crystallinity index
DSC	Differential scanning calorimetry	FWHM	Full width at half maximum
ICP-OES	Inductively coupled plasma optical emission spectroscopy	G′	Storage modulus
D	Diffusion coefficient	G″	Loss modulus
Τ	Tortuosity	tan δ	Loss tangent
Kd	Partition coefficient	E	Elastic/Young’s modulus
ζ	Zeta potential	Q	Swelling ratio
pI	Isoelectric point	EWC	Equilibrium water content
ρx	Crosslink density	TYLCV	Tomato yellow leaf curl virus
EE	Encapsulation efficiency	LCA	Life cycle analysis
Dh	Hydrodynamic diameter	dS m^−1^	Deci-Siemens per meter (electrical conductivity)
Tg	Glass transition temperature	CME	Clathrin mediated endocytosis
Tm	Melting temperature	EDTA	Ethylenediaminetetraacetic acid
N/P ratio	Nitrogen to phosphate ratio	EDDHA	Ethylenediamine-N, N′-bis (2-hydroxyphenylacetic acid)
NPK	Nitrogen, phosphorus, potassium	TMV	Tobacco mosaic virus
